# A Novel Gully-like Surface of Stainless-Steel Fiber Coated with COF-TPB-DMTP Nanoparticles for Solid-Phase Microextraction of Phthalic Acid Esters in Bottled Tea Beverages

**DOI:** 10.3390/nano15050385

**Published:** 2025-03-02

**Authors:** Yuanyuan Yuan, Baohui Li, Keqing Zhang, Hongtao Zhu

**Affiliations:** 1 Department of Environmental Science and Engineering, North China Electric Power University, Baoding 071003, China; yynkyda@163.com (Y.Y.);; 2 Hebei Key Laboratory of Power Plant Flue Gas Multi-Pollutants Control, Baoding 071003, China

**Keywords:** covalent organic frameworks, solid-phase microextraction, gully-like surface, phthalic acid esters, gas chromatography, beverages

## Abstract

A covalent organic framework TPB-DMTP was physically coated onto the gully-like surface of stainless-steel fiber. The fabricated TPB-DMTP-coated stainless-steel fiber was used to extract five phthalic acid esters (PAEs) prior to the GC-FID separation and determination in bottled tea beverages. The developed SPME-GC-FID method gave limits of detection (S/N = 3) from 0.04 µg·L^−1^ (DBP) to 0.44 µg·L^−1^ (BBP), with the enrichment factors from 268 (DEHP) to 2657 (DPP). The relative standard deviations (RSDs) of the built method for inter-day and fiber-to-fiber were 4.1–11.8% and 2.3–9.9%, respectively. The prepared TPB-DMTP-coated stainless-steel fibers could stand at least 180 cycles without a significant loss of extraction efficiency. The developed method was successfully applied for the determination of trace PAEs in different bottled tea beverages, with recoveries from 85.5% to 115%.

## 1. Introduction

Phthalic acid esters (PAEs), a class of organic compounds produced by the esterification of phthalic anhydride with branched monohydric alcohols, are widely used as plasticizers, solvents and additives in various industries, such as food [1], toys [2], cosmetics [3], household buildings [4] and medical devices [5]. As a typical class of endocrine disruptors, PAEs can be transported, migrated and enriched along the food chain through various environmental media, and biomagnified at different trophic levels in the ecosystem [6,7,8]. Since PAEs are bound to biomolecules in the body via non-covalent forms (van der Waals forces and hydrogen bonding forces) while circulating through humans, PAEs can lead to teratogenic, carcinogenic and mutagenic effects [9,10]. In 1991, dimethyl phthalate (DMP), diethyl phthalate (DEP), dibutyl phthalate (DBP), butyl benzyl phthalate (BBP), dioctyl phthalate (DNOP) and di(2-ethylhexyl) phthalate (DEHP) were listed as the priority pollutants by the Environmental Protection Agency [11]. The limits of DEP, DBP and DEHP in Chinese drinking water quality are 0.3 mg·L^−1^, 0.003 mg·L^−1^ and 0.008 mg·L^−1^, respectively [12]. Since gas chromatography (GC) has the advantages of high sensitivity, good separation and high efficiency, it is suitable for the detection of volatile or semi-volatile organic compounds, such as PAEs, whose boiling points are 180–500 °C [13,14].

In view of the low concentration of PAEs in complex matrices, it is necessary to select an appropriate pretreatment to improve the pre-column concentration of PAEs. As a convenient, fast and green sample pretreatment technique that integrates collection, extraction, desorption and injection into one step, solid-phase microextraction (SPME) was developed by Pawliszyn and Arthur in 1990 [15], which has been widely employed in various fields, such as food [16], biomedicine [17] and the environment [18]. SPME usually consists of a substrate and a coating that mainly includes the physical coating mode and the chemical grafting one. The traditional fiber substrate is made of quartz, which is easily broken [19]. Therefore, stainless-steel [20], titanium [21], copper foam [22] and aluminum [23] substrate fibers were explored to overcome the disadvantage of quartz fiber. Furthermore, a variety of coating materials were explored to expand the applications of the metal fibers, including carbon nanotubes [24], metal organic frameworks [25,26,27], covalent organic frameworks (COFs) [28,29,30], ionic liquids [31,32,33] and molecularly imprinted polymers [34,35].

COFs, discovered by Yaghi [36] in 2005, are porous organic materials composed of ordered organic monomers. Owing to their merits of excellent thermal stability, controllable size and great chemical stability, COFs have been widely used in gas adsorption [37,38], energy storage and conversion [39], pollutant removal [40], sensing [41], catalysis [42], etc. Currently, a variety of COFs are employed as the coatings of SPME fibers. Wang and his co-workers [43] developed COF-immobilized SPME fibers by chemically bonding SNW-1 on stainless-steel fiber, while the prepared fiber was used to extract phenolic compounds from honey samples with limits of detection from 0.06 ng·g^−1^ to 0.2 ng·g^−1^.

However, no matter how the coating was applied to the metal fiber (i.e., physical mode or chemical mode), the coatings of the metal fiber were easily exfoliated due to the repeated thermal expansions and contractions and the friction between the coating and the syringe during the extraction procedure. Consequently, the metal fiber coating could suffer from worse reproducibility and a shorter service time. To solve this problem, some researchers began to create fibers with rough surfaces via chemical and electrochemical etching methods [44,45,46]. Li and his co-workers [20] fabricated one kind of robust coating fiber to improve the service time and extraction efficiency via binding COF-TpBD into the arrayed nanopores of stainless-steel fibers.

In this study, the COF-TPB-DMTP was physically filled into the gully-like surface of SSF prepared with a two-step electrochemical etching method. The TPB-DMTP-coated gully-like surface SSF was used to pretreat the PAEs in a bottled tea beverage prior to the GC-FID separation and detection. For only the physical coating, the prepared TPB-DMTP-GS-SSF exhibited a sound service time and reproducibility thanks to the firm combination between the COFs coating and the gully-like surface of the SSF.

## 2. Materials and Methods

### 2.1. Chemicals and Reagents

All chemicals and reagents used in this experiment were at least analytical grade without further purification. Ammonium fluoride (≥96.0%), methanol (99.9%), ethylene glycol (≥99.0%), anhydrous ethanol (≥99.7), acetonitrile (≥99.9) and sodium chloride were from the Tianjin Kermel Chemical Reagent Co., Ltd. (Tianjin, China). Acetic acid (99.5%) was bought from the Shanghai Macklin Biochemical Co., Ltd. (Shanghai, China). Acetone (≥99.0%) was from the Tianjin Damao Chemical Reagent Factory (Tianjin, China). Perchloric acid (≥70.0%) was obtained from the Xilong Scientific Co., Ltd. (Shenzhen, China). Octadecane (≥98.0%), eicosane (≥99.0%) and heneicosane (≥99.0%) were obtained from the Aladdin Chemical Reagent Co., Ltd. (Shanghai, China). Nitrogen (N_2_) was supplied by the Hanjiangxue Trading Co., Ltd. (Baoding, China). 1,3,5-tris(aminophenyl)benzene (TPB), 2,5-dimethoxyterephthalaldehyde (DMTP), dibutyl phthalate (DBP, ≥99.0), diisobutyl phthalate (DIBP, 99%), benzyl butyl phthalate (BBP, ≥98%), dipentyl phthalate (DPP, 95%) and di(2-ethylhexyl) phthalate (DEHP, ≥98%) were from the Shanghai Dibai Biotechnology Co., Ltd. (Shanghai, China). Each PAE (10 mg) was dissolved in methanol and diluted into the 10 mL volumetric flask to make the 1000 mg·L^−1^ stock solution, which was stored at 4 °C in the dark. Ultrapure water collected from a Milli-Q integral system (Millipore China Co., Ltd., Shanghai, China) was used throughout this experiment. Bottled green tea, jasmine tea and oolong tea beverages used in this experiment were purchased from the local supermarket (Baoding, China).

### 2.2. Instrumentation

The GC 2014C gas chromatograph with FID was purchased from the Shimadzu Co. (Kyoto, Japan). The HP-5 chromatographic column (30 m × 0.32 mm ID × 0.5 µm) was obtained from the Lanzhou Institute of Chemical Physics (Lanzhou, China). The DP800A series programmable linear DC power supply was sourced from the Beijing Puyuanjingdian Technology Co., Ltd. (Beijing, China). The 5 µL syringes were from the Shanghai Gaoge Industrial and Trade Co., Ltd. (Shanghai, China). Commercial SPME fibers (PDMS (30 μm) and DVB/CAR/PDMS (50/30 μm)) were procured from the Sigma-Aldrich Trading Co., Ltd. (Shanghai, China).

The Fourier-transform infrared (FT-IR) spectra were measured on a Tensor II (Bruker AXS GmbH, Karlsruhe, Germany). The thermogravimetric analysis (TGA) was performed on a TGA 4000 thermal gravimetric analyzer (Rigaku, Tokyo, Japan). The scanning electron microscope (SEM) images were recorded on a Hitachi S4800 (Hitachi, Tokyo, Japan). The X-ray diffraction (XRD) data were obtained using a D8 Advance diffractometer (Bruker AXS GmbH, Karlsruhe, Germany).

### 2.3. The Chromatographic Conditions

The chromatographic conditions were set as follows: High-purity N_2_ was used as the carrier gas with a 30 mL∙min^−1^ flow rate. The flow rates of H_2_ and air were 40 mL∙min^−1^ and 400 mL∙min^−1^, respectively. The injector and detector temperatures were set at 280 °C and 300 °C, respectively. The column temperature was held at 120 °C for 1 min, increased to 220 °C at a rate of 20 °C∙min^−1^, then increased to 280 °C at 5 °C∙min^−1^ and kept for 2 min. The total run time was 18 min and all injections were carried out in the splitless mode.

### 2.4. The Preparation of the COF-TPB-DMTP Used for Characterization

The COF-TPB-DMTP was synthesized according to the literature with a minor modification (see Figure S1) [47]. TPB (126.5 mg) and DMTP (93.2 mg) were dissolved in a liner that contained 30 mL of acetonitrile and 0.5 mL of acetic acid. This liner was transferred to a magnetically stirred hydrothermal synthesis reactor at 250 rpm, heated to 90 °C at a rate of 1.5 °C·min^−1^ and kept for 24 h, then cooled down to 30 °C at a rate of 1.5 °C·min^−1^. Finally, the orange solid of COF-TPB-DMTP was obtained by washing with anhydrous ethanol and acetone.

### 2.5. The Fabrication of the Gully-like Surface of the SSF

To remove the organic compounds adhered to the surface of the SSF, the polished 304 SSF with sandpaper were ultrasonicated with acetone, ethanol and ultrapure water for 15 min each. As the anode of the etching device, the rinsed SSF was placed into the center line of the graphite tube, which was employed as a cathode. A two-step etching method was used to prepare the gully-like surface of SSF. In the first step, the 3 cm length bottom of the SSF was etched at 25 V with a mixture of anhydrous ethylene glycol and perchloric acid (V/V, 9:1) as the electrolyte. In the second step, the SSF was etched for 5 min at 50 V with a mixture solution of ethylene glycol that contained 0.1 mol·L^−1^ ammonium fluoride as the electrolyte. After being rinsed with ultrapure water, the gully-like SSF (GS-SSF) was obtained. The comparison between different etching methods for the preparation of SPME fibers is summarized in Table 1.

### 2.6. The Fabrication of the COF-TPB-DMTP-Coated SPME Fibers

**Figure 1:**
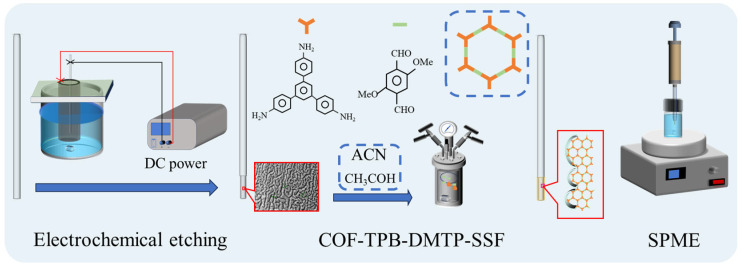
The schematic diagram of the fabrication process for the TPB-DMTP-GS-SSF.

The etched bottom of the GS-SSF was vertically immersed in a magnetically stirred hydrothermal synthesis reactor that contained 30 mL acetonitrile, 0.5 mL acetic acid, and certain amounts of TPB and DMTP. The COF-TPB-DMTP was synthesized into the gullies of the stainless-steel fiber in situ according to Section 2.4. Then, the dried fiber was loaded into a 5 µL syringe and aged at 300 °C for 6 h. The preparation process of TPB-DMTP-GS-SSF is shown in Figure 1.

### 2.7. SPME Procedure

All extractions were conducted using direct immersion SPME mode. The treated TPB-DMTP-GS-SSF was directly immersed into a 20 mL working solution in an extraction vial for the 30 min extraction. Then, the fiber was removed from the extraction vial rapidly and immediately transferred to the GC injection port for desorption at 280 °C for 2 min.

## 3. Results

### 3.1. The Characterization of the COF-TPB-DMTP and COF-TPB-DMTP Coating

**Figure 2:**
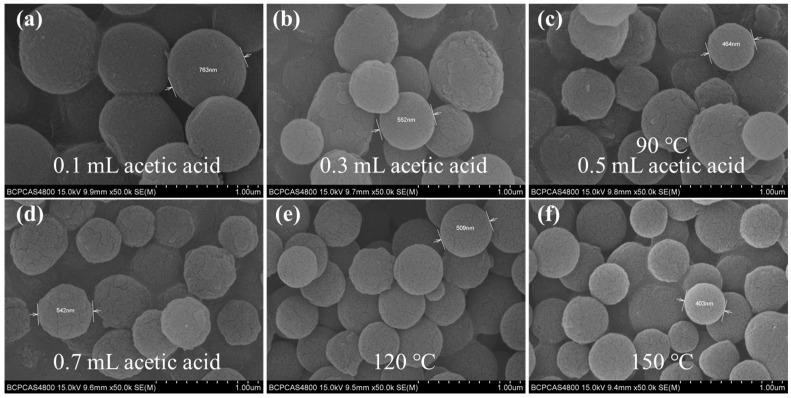
The SEM images of COF-TPB-DMTP synthesized under different conditions with (**a**–**d**) acetic acid amounts of 0.1, 0.3, 0.5 and 0.7 mL at 90 °C and (**e**,**f**) 0.5 mL of acetic acid at 120 °C and 150 °C.

Considering the gully-like size of the GS-SSF, it was essential to control the diameter of the coating material. The parameters that influenced the sizes of the COFs included the proton number, reaction concentration and temperature. When the amount of acetic acid was varied from 0.1 mL to 0.5 mL at 90 °C, the spherical diameter of the COF-TPB-DMTP decreased gradually (Figure 2a–d). This could be attributed to the promotion of nucleophilic attack behavior with the increase in the amount of acid. However, an excessive acetic acid concentration could lead to N-protonation, which could reduce or eliminate the nucleophilicity and hinder the nucleophilic attack behavior. In addition, the influence of the reaction temperature on the morphology structure of the COF-TPB-DMTP was also investigated. As shown in Figure 2c,e,f, when the temperature varied from 90 °C to 150 °C, the diameter of the spherical COF remained almost unchanged.

**Figure 3:**
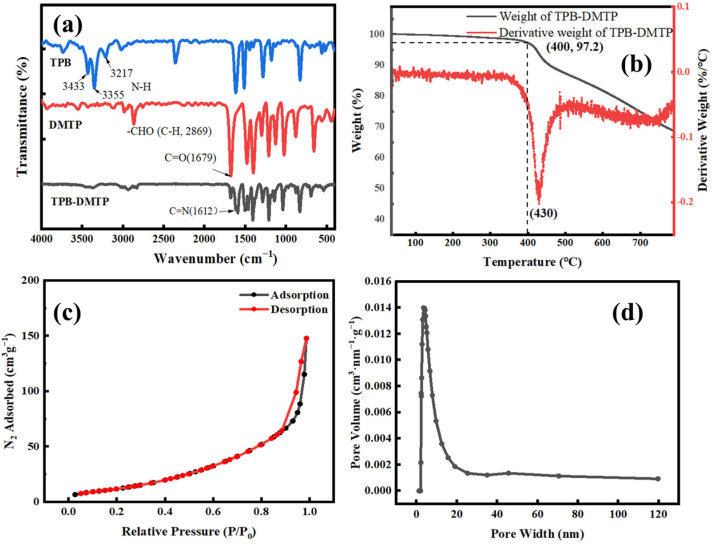
(**a**) The FT-IR spectra of the powdered COF-TPB-DMTP; (**b**) the TGA curve of the powdered COF-TPB-DMTP; (**c**) the N_2_ sorption–desorption isotherms of the powdered COF-TPB-DMTP; (**d**) the pore size distribution of the powdered COF-TPB-DMTP.

The appearance of C=N stretching bands at 1612 cm^−1^ illustrated the successful synthesis of COF-TPB-DMTP since the C=N was nonexistent in the TPB and DMTP (Figure 3a). The disappearance of the N-H stretching bands (3433, 3355 and 3217 cm^−1^) and the weakening of the -CHO stretching bands (1679 and 2869 cm^−1^) of COF-TPB-DMTP in comparison with those of the TPB and DMTP also suggest the Schiff base condensation reaction had finished, which agreed with previously reported results [57]. The TGA revealed that the weight loss (<3%) of the prepared COFs was not obvious when the temperature was below 400 °C (Figure 3b). This result indicates that the resultant COF-TPB-DMTP possessed high thermal stability. The porosity and surface area of the prepared COF-TPB-DMTP were evaluated by nitrogen adsorption–desorption measurements at 77 K (Figure 3c). The type-III adsorption isotherm indicated the existence of a mesoporous structure in the COF-TPB-DMTP. The specific surface area, pore volume and pore diameter of the resultant COF-TPB-DMTP were calculated to be 81.01 m^2^·g^−1^, 0.243 cm^3^·g^−1^ and 3.429 nm, respectively. The abundant mesoporous structure is seen in Figure 3d.

**Figure 4:**
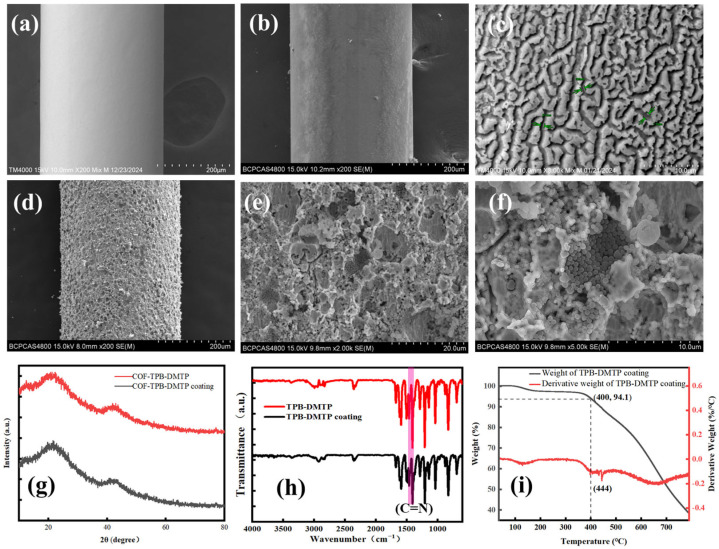
(**a**) The SEM images of the 304 SSF at 200×; (**b**,**c**) the SEM images of the 304 stainless steel after etching (**b**) at 200× and (**c**) at 3000×; (**d**–**f**) the SEM images of the TPB-DMTP-GS-SSF (**d**) at 200×, (**e**) at 2000× and (**f**) at 5000×; (**g**) the XRD images of the COF-TPB-DMTP versus the COF-TPB-DMTP-GS-SSF; (**h**) the FT-IR image of the COF-TPB-DMTP and TPB-DMTP-GS-SSF; (**i**) the TGA image of the COF-TPB-DMTP and TPB-DMTP-GS-SSF.

The SEM image (Figure 4a–c) of the GS-SSF exhibited a distinct gully-like surface structure. The nano-sized spherical COF-TPB-DMTP particles coated the gullies (Figure 4d–f). In comparison with the XRD pattern of the COF-TPB-DMTP (Figure 4g), one of the COF-TPB-DMTP coatings scraped from the prepared fiber was not altered remarkably. The results revealed that the crystallinity of the COF-TPB-DMTP fabricated with two approaches was identical. In fact, the FT-IR spectra of the COF-TPB-DMTP and COF-TPB-DMTP coatings could give the same result (see Figure 4h). The TPB-DMTP coating material possessed a superior thermal stability and could be combined with GC (Figure 4i).

### 3.2. The Optimization of the Extraction Conditions

**Figure 5:**
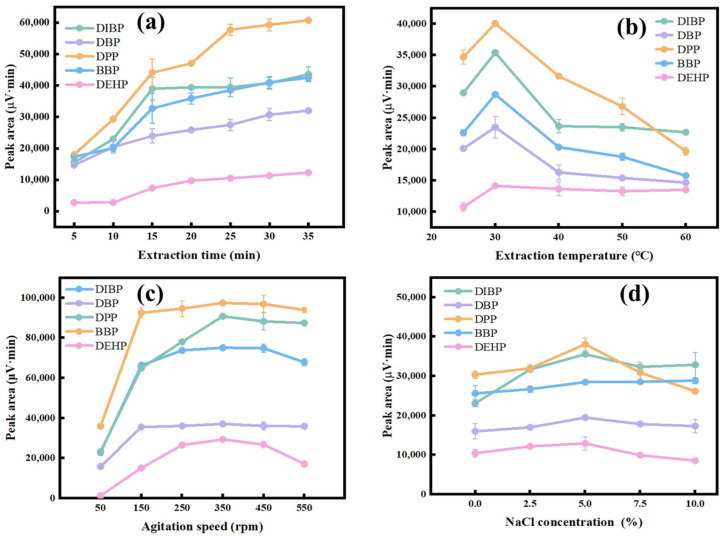
The effects of extraction parameters on the extraction efficiency for the PAEs: (**a**) extraction time (conditions: extraction temperature, 30 °C; desorption time, 2 min; desorption temperature, 280 °C; NaCl concentration, 5%; agitation speed, 250 rpm); (**b**) extraction temperature (conditions: extraction time, 30 min; desorption time, 2 min; desorption temperature, 280 °C; NaCl concentration, 5%; agitation speed, 250 rpm); (**c**) agitation speed (conditions: extraction time, 30 min; extraction temperature, 30 °C; desorption time, 2 min; desorption temperature, 280 °C; NaCl concentration, 5%); (**d**) NaCl concentration (conditions: extraction time, 30 min; extraction temperature, 30 °C; desorption time, 2 min; desorption temperature, 280 °C; agitation speed, 250 rpm). The extraction solution contained DIBP 30 µg·L^−1^, DBP and DPP 10 µg·L^−1^, and BBP and DEHP 50 µg·L^−1^.

Various parameters that influenced the extraction efficiency of the SPME of PAEs on the COF-TPB-DMTP coated fiber, such as the extraction temperature, extraction time, agitation speed and ionic strength, were optimized. SPME is a dynamic equilibrium extraction technique, so the number of adsorbed analytes increases with the lengthening of the extraction time until dynamic equilibrium is reached [58]. The effect of the extraction time was optimized from 5 to 35 min (Figure 5a). The peak areas of the PAEs rapidly increased with the variation in the extraction time from 5 min to 30 min. To achieve the best extraction efficiency, 30 min was chosen as the extraction time.

The higher temperature could facilitate the diffusion of analytes, which enabled shortening the time that the analytes required to reach the adsorption equilibrium. However, an excessive temperature could decrease the distribution coefficient of PAEs between the coating and the sample matrix, which could result in a drastic drop in the extraction efficiency. The effect of the extraction temperature was examined from 25 °C to 60 °C (Figure 5b). It can be seen that the extraction efficiency dropped sharply when the extraction temperature was over 30 °C. Therefore, 30 °C was chosen as the optimized extraction temperature in the further studies.

The effect of the agitation speed was evaluated from 50 rpm to 550 rpm (Figure 5c). The results show that the peak areas of the five PAEs increased when the agitation speed varied from 50 rpm to 250 rpm. Once the agitation speed was over 250 rpm, a reduction in the extraction efficiency was observed. This could be attributed to the appearance of the vortex, which could lead to the removal of the fiber from the sample matrix. So, an agitation speed of 250 rpm was chosen in the following experiments.

Since the ionic strength could cause a variation in the viscosity, the effect of the ionic strength was discussed, which was replaced with a certain concentration of NaCl (Figure 5d). With the concentration of NaCl up to 5%, the peak areas of the five PAEs increased due to the “salting-out” effect. Instead, the extraction efficiencies showed a negative result with the further improvement of the concentration of NaCl due to the increased viscosity, which could hinder the diffusion of PAEs [20]. Therefore, 5% NaCl concentration was added into the sample metrics for the SPME.

### 3.3. Optimization of the Desorption Conditions

**Figure 6:**
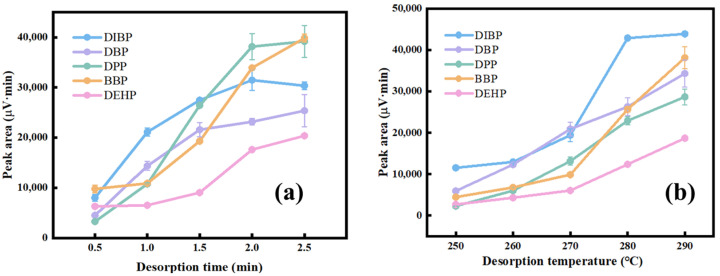
The effects of extraction parameters on the desorption efficiency for the PAEs: (**a**) desorption time (conditions: extraction time, 30 min; extraction temperature, 30 °C; desorption temperature, 280 °C; NaCl concentration, 5%; agitation speed, 250 rpm); (**b**) desorption temperature (conditions: extraction time, 30 min; extraction temperature, 30 °C; desorption time, 2 min; NaCl concentration, 5%; agitation speed, 250 rpm). The extraction solution contained DIBP 30 µg·L^−1^, DBP and DPP 10 µg·L^−1^, and BBP and DEHP 50 µg·L^−1^.

In the desorption procedure, the desorption time and the desorption temperature are critical factors that influenced the extraction efficiency. At first, the effect of the desorption time from 0.5 min to 2.5 min was examined (Figure 6a). The results show that the extraction efficiency increased continuously with the increase in the desorption time. However, a too long desorption time resulted in the peak broadening of PAEs. So, a 2 min desorption time was chosen for the further experiments. Second, the effect of the desorption temperature was evaluated from 260 °C to 290 °C (Figure 6b). The higher the temperature, the higher the peak area of the five PAEs. Considering the maximum temperature tolerance of the HP-5 column, 280 °C was selected as the desorption temperature.

### 3.4. The Analytical Figures of Merit

**Figure 7:**
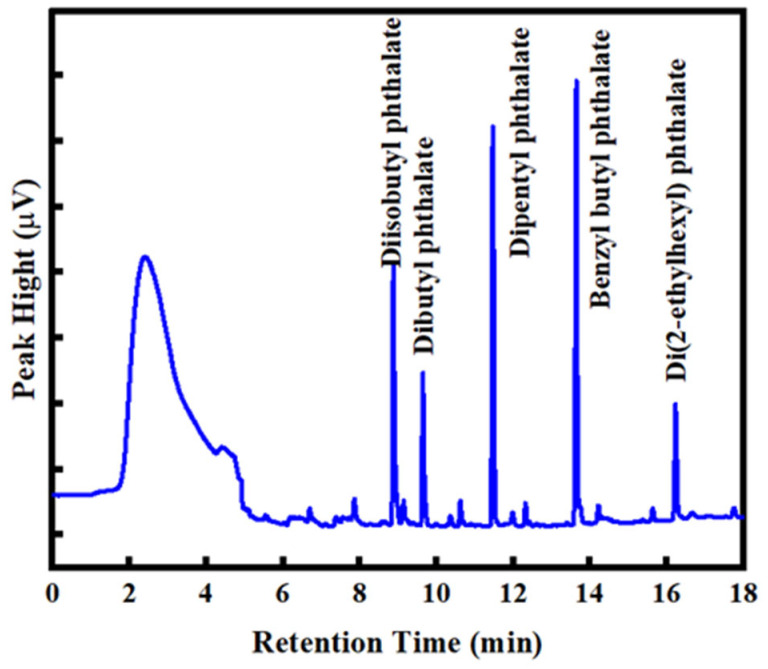
The chromatogram of the five PAEs in the optimized conditions. Extraction solution: a mixture that contained DIBP 30 µg·L^−1^, DBP and DPP 10 µg·L^−1^, and BBP and DEHP 10 µg·L^−1^. The extraction conditions: extraction time, 30 min; extraction temperature, 35 °C; agitation speed, 250 rpm; salt concentration, 5%. The desorption conditions: desorption time, 2 min; desorption temperature 280 °C.

The chromatogram obtained under the optimized conditions (Figure 7) shows the successful separation and detection of the five PAEs, namely, DBP, DIBP, BBP, DPP and DEHP, in water. The analytical figures of merit of the prepared COF-TPB-DMTP-coated fiber for the SPME of PAEs are summarized in Table 2, namely, the linear range, correlation coefficient (R^2^), limits of detection (LODs), limits of quantification (LOQs), inter-day and fiber-to-fiber relative standard deviations (RSDs), and enrichment factors (EFs). The EFs for the PAEs ranged from 268 to 2657. The LODs (S/N = 3) and LOQs (S/N = 10) were in the ranges of 0.04–0.44 µg·mL^−1^ and 0.13–1.46 µg·mL^−1^, respectively. The linear range for the PAEs varied from 0.04 µg·mL^−1^ to 500 µg·mL^−1^. The inter-day RSDs for the five days of replicate extractions of the PAEs ranged from 4.1% to 12.9%. The intra-day RSDs of the PAEs repeatedly extracted during the three periods of the day ranged from 3.9% to 14.1%. The RSDs for fiber-to-fiber obtained on three parallelly prepared fibers were less than 9.9%. In addition, the TPB-DMTP-GS-SSF showed a high enrichment efficiency for DIBP, DBP, DPP and BBP in water.

**Figure 8:**
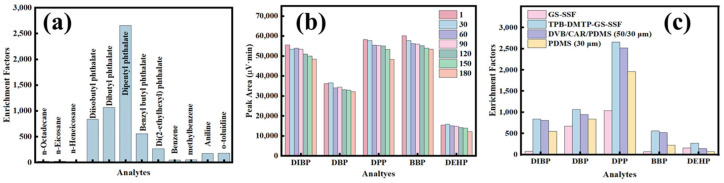
(**a**) Analyte selectivity analysis of the TPB-DMTP-GS-SSF; (**b**) the service time of the TPB-DMTP-GS-SSF; (**c**) comparison of the TPB-DMTP-GS-SSF with commercial SPME fibers. The extraction solution contained DIBP 30 µg·L^−1^, DBP and DPP 10 µg·L^−1^, and BBP and DEHP 50 µg·L^−1^.

The adsorption selectivity of the TPB-DMTP-GS-SSF was tested by the extraction of several organic compounds with different polarities and conjugation effects, including n-octadecane, n-eicosane, n-heneicosane, DIBP, DBP, DDP, BBP, DEHP, benzene, methylbenzene, aniline and o-toluidine (Figure 8a). It can be seen that the TPB-DMTP-GS-SSF offered excellent extraction efficiency and selectivity for the PAEs. The EF of aniline and o-toluidine was higher than those of the straight chain alkanes, benzene and toluene. The phenomena could be attributed to the π-π stacking interactions on the benzene ring. The results highlight the potential of the TPB-DMTP-GS-SSF for the extraction of the polar organic compounds. As shown in Figure 8b, no significant loss of extraction efficiency for the PAEs was observed on the COF-TPB-DMTP-coated fiber even after 180 cycles of extraction/desorption.

In order to verify the selectivity and the extraction efficiency of the prepared TPB-DMTP-GS-SSF, two kinds of commercial SPME fibers (PDMS and DVB/CAR/PDMS coating fibers), the GS-SSF and the TPB-DMTP-GS-SSF were also employed to extract the PAEs in water (Figure 8c). The TPB-DMTP-GS-SSF gave sound EFs from 268 to 2657, which were higher than the ones of the GS-SSF from 69 to 1036, the ones of the PDMS fiber from 72 to 1958 and the ones of DVB/CAR/PDMS fiber from 141 to 2516. For a further comparison of the enrichment capacity, the average percentage increase calculations were carried out and the results shown in Table S1. It was found that all the average percentage increases in the EF of the TPB-DMTP-GS-SSF were over 4.46%. The above results reveal the significant potential of the prepared TPB-DMTP-GS-SSF for the extraction of PAEs. A summary of the comparative study of the developed method with other reported methods for the determination of the PAEs in various samples are shown in Table 3.

### 3.5. Analysis of PAEs in Commercially Bottled Tea Samples

The developed SPME-GC-FID method was employed for the determination of trace PAEs in bottled tea beverages (Table 4). The recoveries ranged from 85.5% to 115%, obtained by spiking the standard PAEs solution in the bottled tea beverages, which corresponded to spiking 30 µg·L^−1^ DIBP, 10 µg·L^−1^ DBP, 10 µg·L^−1^ DPP, 50 µg·L^−1^ BBP and 50 µg·L^−1^ DEHP in a working solution. The recoveries ranged from 86.53% to 106.07%, which were obtained by spiking the standard PAEs solution, which corresponded to spiking 15 µg·L^−1^ DIBP, 5 µg·L^−1^ DBP, 5 µg·L^−1^ DPP, 25 µg·L^−1^ BBP and 25 µg·L^−1^ DEHP in a working solution.

## 4. Conclusions

In summary, a high-efficiency and long-service-time GS-SSF physically coated with COF-TPB-DMTP was developed, which was etched via the two-step electrochemical etching method. The built SPME-GC-FID method was employed to determine five PAEs in bottled tea beverages. The TPB-DMTP-GS-SSF showed larger EFs than the bare GS-SSF and the two commercial fibers. For only the physical coating, the prepared TPB-DMTP-GS-SSF exhibited a sound service time, low LODs and reproducibility due to the firm combination between the COFs coating and the gully-like surface of the SSF.

## Figures and Tables

**Table 1:** The comparison between different etching methods for the preparation of the SPME fibers.

Etched Methods	Etched Solution	Time	Coating	Analytics	Ref.
Chemical etching	1 M NaOH	30 min	Carbon-aerogel-based	OPs	[48]
Chemical etching	HF (40%)	20 min	Ionic liquids	Alkylphenols	[49]
Chemical etching	HF (40%)	-	Carbonized MXene-polyvinylpyrrolidone	PAHs	[50]
Polished	-	-	Poly(1-Hexyl-3-Vinylimidazolium) Bromide	PCBs	[51]
Chemical etching	0.1 M NaOH and 0.1 M HCl	Washed	Ionic liquids	OPEs	[52]
Chemical etching	H_2_SO_4_	-	MOF/PANI nanocomposites	Aromatic compounds	[53]
Chemical etching	FeCl_3_/HCl solution (20% *w*/*v*)	15 min	-	PAHs	[54]
Chemical etching	Aqua regia/water (1:1)	30 min	Montmorillonite/ionic liquid composite	Phenolic compounds	[55]
Chemical etching	37% HCl	30 min	Multiple-helix cobalt (II)-based Metal–organic nanotube	Chlorophenol and nitrophenols	[56]
Electrochemical etching	(CH_2_OH)_2_/HClO_4_ (*v*/*v*, 9:1)	50 s	COF-TpBD	PAHs	[20]
Electrochemical etching	(CH_2_OH)_2_/HClO_4_ (*v*/*v*, 9:1); 0.1 M (CH_2_OH)_2_ of NH_4_F	6 min	COF-TPB-DMTP	PAEs	This work

**Table 2:** The analytical performance of the TPB-DMTP-GS-SSF for the SPME of the PAEs.

Analytes	Linear Range (µg·L^−1^)	R^2^	LODs (µg·L^−1^)	LOQs (µg·L^−1^)	RSDs					EFs *
					Inter-Day (n = 5, %)		Intra-Day (n = 3, %)		Fiber-to-Fiber (n = 3, %)	
					a	b	a	b		
DIBP	0.20–300	0.9932	0.20	0.67	4.1	9.3	4.3	8.7	8.4	840
DBP	0.04–100	0.9964	0.04	0.13	8.0	5.2	6.3	3.8	9.9	1068
DPP	0.30–100	0.9973	0.30	0.99	11.8	12.1	7.3	12.3	4.5	2657
BBP	0.44–500	0.9928	0.44	1.46	9.1	12.9	7.9	11.9	2.3	559
DEHP	0.39–500	0.9945	0.39	1.30	10.5	12.5	3.9	14.1	7.5	268

* The enrichment factor (EF) for this experiment was calculated using EF = $\frac{S_{SPME}\times 100}{S_{DI}}$. *S_DI_* refers to the peak area obtained by the direct injection of 1 µL of a mixture that contained DIBP 3000 µg·L^−1^, DBP and DPP 1000 µg·L^−1^, and BBP and DEHP 5000 µg·L^−1^; *S_SPME_* refers to the peak area measured using the SPME-GC method from a 20 mL mixture that contained 30 µg·L^−1^ DIBP, 10 µg·L^−1^ DBP and DPP, and 50 µg·L^−1^ BBP and DEHP. a: 30 µg·L^−1^ DIBP, 10 µg·L^−1^ DBP, 10 µg·L^−1^ DPP, 50 µg·L^−1^ BBP and 50 µg·L^−1^ DEHP. b: 15 µg·L^−1^ DIBP, 5 µg·L^−1^ DBP, 5 µg·L^−1^ DPP, 25 µg·L^−1^ BBP and 25 µg·L^−1^ DEHP.

**Table 3:** The comparison of the developed method with other reports on the extraction of PAEs.

Method	Coating	Matrix	Analytes	Linear Range (µg·L^−1^)	LODs (µg·L^−1^)	Ref.
HPLC-UV	ZIF-8-90@graphene oxide	Water, juice	DMP, DEP, DBP	0.1–500	0.026–0.058	[59]
GC-MS	MWNTs@PS	water	DBP, BBP, DEHP, DOP	0.001–5	0.0012–0.018	[24]
GC-MS	HCP-1	Bottled water	DMP, DEP, DAP, DPRP, DIBP, DBP, BBP, DCHP	0.01–10	0.003–0.033	[60]
GC-MS-MS	TpBD-TiO_2_	Vegetables	DMP, DEP, DIBP, DBP, DMPP, DPP, DHXP, BBP, DCHP, DPhP, DNOP	0.005–50	0.001–0.430	[29]
GC-FID	MNP@P3TArH	Bottled mineral and fresh milk	DMP, DEP, DPP, DBP, BBP, DCP, DEHP, DNOP	0.1–50	0.054–0.468	[61]
GC-FID	OH_50%_-TPB-COF	Water	DEP, DPP, DAP, DBP, BBP, DEHP	1–100	0.032–0.451	[62]
GC-FID	COF-TPB-DMTP	Bottled tea beverages	DIBP, DBP, BBP, DPP, DEHP	0.04–500	0.04–0.44	This work

**Table 4:** The analytical results for the determination of the PAEs in commercially bottled tea samples.

Analytes	Bottled Green Tea			Jasmine Tea			Oolong Tea		
	Found (µg L^−1^)	Recoveries (%, n = 3)		Found (µg L^−1^)	Recoveries (%, n = 3)		Found (µg L^−1^)	Recoveries (%, n = 3)	
		Added ^a^	Added ^b^		Added ^a^	Added ^b^		Added ^a^	Added ^b^
DIBP	ND	106	87.57	ND	115	95.54	ND	94.3	91.00
DBP	ND	85.5	106.07	ND	100	92.86	ND	89.3	90.18
DDP	ND	89.4	98.37	ND	79.0	96.47	ND	108	86.53
BBP	ND	108	97.78	ND	90.0	102.73	ND	80.2	97.94
DEHP	ND	115	98.10	ND	114	96.37	ND	90.4	95.79

ND: not detected. ^a^: the spiked concentrations were 30 µg·L^−1^ DIBP, 10 µg·L^−1^ DBP, 10 µg·L^−1^ DPP, 50 µg·L^−1^ BBP and 50 µg·L^−1^ DEHP in a working solution. ^b^: the spiked concentrations were 15 µg·L^−1^ DIBP, 5 µg·L^−1^ DBP, 5 µg·L^−1^ DPP, 25 µg·L^−1^ BBP and 25 µg·L^−1^ DEHP in a working solution.

## Data Availability

Data are contained within the article and Supplementary Materials.

## Supplementary Materials

The following supporting information can be downloaded at: https://www.mdpi.com/article/10.3390/nano15050385/s1, Figure S1. The schematic diagram of the synthesis of COF-TPB-DMTP; Table S1 Average percentage increase in EFs of the TPB-DMTP-GS-SSF in five PAEs with different fibers.

## Abbreviations

The following abbreviations are used in this manuscript:

COFs	Covalent organic frameworks
PAEs	Phthalic acid esters
SPME	Solid-phase microextraction
SSF	Stainless-steel fiber
GS-SSF	Gully-like surface of stainless-steel fiber
TPB	1,3,5-tris(aminophenyl)benzene
DMTP	2,5-dimethoxyterephthalaldehyde
DBP	Dibutyl phthalate
DIBP	Diisobutyl phthalate
BBP	Benzyl butyl phthalate
DPP	Dipentyl phthalate
DEHP	Di(2-ethylhexyl) phthalate
GC	Gas chromatography
FID	Flame ionization detector
FT-IR	Fourier-transform infrared
TGA	Thermogravimetric analysis
SEM	Scanning electron microscope
XRD	X-ray diffraction
LODs	Limits of detection
LOQs	Limits of quantification
RSDs	Relative standard deviations
EFs	Enrichment factors
ILs	Ionic liquids
MOFs	Metal–organic frameworks
GC-MS	Gas chromatography–mass spectrometry
OPs	Organophosphorus pesticides
PAHs	Polycyclic Aromatic Hydrocarbons
PCBs	Polychlorinated Biphenyls
OPEs	Organophosphate Esters
