# Supplementary material for: A Novel Gully-like Surface of Stainless-Steel Fiber Coated with COF-TPB-DMTP Nanoparticles for Solid-Phase Microextraction of Phthalic Acid Esters in Bottled Tea Beverages
Source: Nanomaterials (Basel). 2025 Mar 2;15(5):385. doi: 10.3390/nano15050385 (PMC11901468; doi:10.3390/nano15050385)
Supplement: Supplementary file 1 [file nanomaterials-15-00385-s001.zip › nanomaterials-3470633-supplementary.pdf]

Supporting information

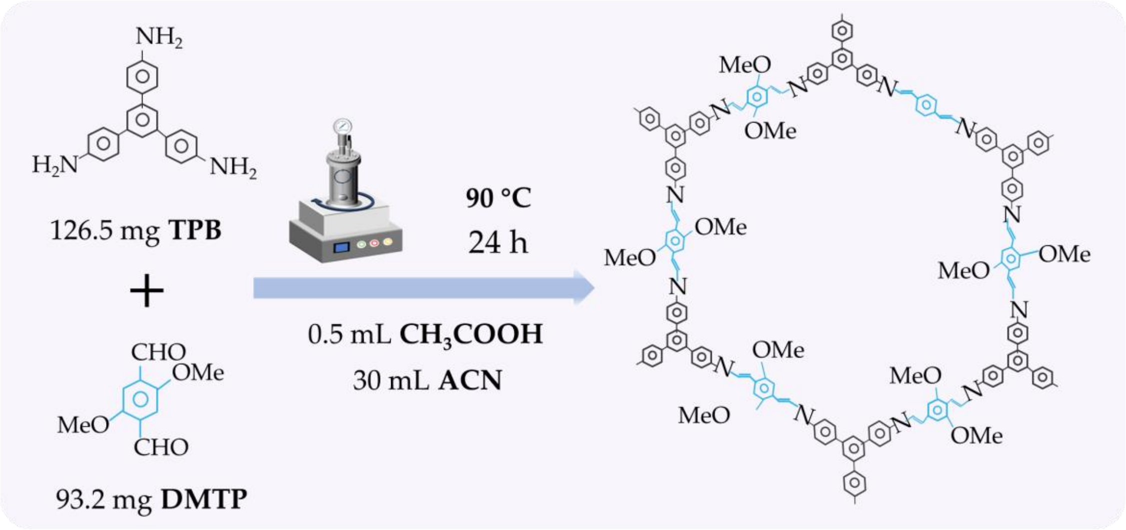

Figure S1. The schematic diagram of the synthesis of COF-TPB-DMTP

Table S1 Average percentage increase in EFs of the TPB-DMTP-GS-SSF in five PAEs with different fibers.

Analytes	DIBP	DBP	DPP	BBP	DEHP
API_1	4.46%	13.25%	5.61%	7.83%	90.15%
API_2	53.01%	27.63%	35.73%	153.57%	273.61%

$$API_1(\text{Average percentage increase 1}) = \frac{EF_{(\text{TPB-DMTP-GS-SSF})} - EF_{(\text{DVB/CAR/PDMS})}}{EF_{(\text{DVB/CAR/PDMS})}};$$

$$API_2(\text{Average percentage increase 2}) = \frac{EF_{(\text{TPB-DMTP-GS-SSF})} - EF_{(\text{PDMS})}}{EF_{(\text{PDMS})}}.$$
